# The ability of Austrian registered physiotherapists to recognize serious pathology

**DOI:** 10.1186/s12875-024-02634-8

**Published:** 2024-10-30

**Authors:** Jessie Janssen, Wolfgang Lackenbauer, Simon Gasselich, Martina Edda Lickel, Lars Schabel, Reinhard Beikircher, Christian Keip, Manfred Wieser, James Selfe, Bruno Mazuquin, Gillian Yeowell

**Affiliations:** 1 Institute of Therapeutic and Midwifery Sciences, Department of Health Sciences, IMC University of Applied Sciences Krems, Krems an der Donau, Austria; 2 Karl Landsteiner University of Health Sciences, Human Medicine, Krems an der Donau, Austria; 3 Clinical Department of Orthopaedics and Traumatology, University Hospital Tulln, Tulln, Austria; 4 Department of Health Professions, Manchester Metropolitan University, Manchester, UK

**Keywords:** Red flags, Physical therapy, Clinical decision making, Clinical vignettes

## Abstract

**Background:**

Serious pathology masking as musculoskeletal conditions is rare, still it is pertinent that physiotherapists can recognise it. This ability has been investigated internationally, however the decision-making skills of registered Austrian physiotherapists has not been examined. The aim of this study was to assess the ability of registered Austrian physiotherapists to make accurate keep-refer decisions based on clinical vignettes.

**Methods:**

In this national survey registered Austrian (self-)employed physiotherapists were recruited and completed 12 clinical vignettes. Correctly answered vignettes were listed as percentages.

**Results:**

479 physiotherapists participated in the study. The response rate of the self-employed physiotherapists was 8.0%. On average participants classified 70.5% of the musculoskeletal cases, 79.4% of the non-critical medical cases, and 53.3% of the critical medical cases correctly.

**Conclusion:**

This study suggests that, despite the limitations of using written clinical vignettes, registered Austrian physiotherapists welcome additional training to improve their skills in identifying serious pathology. Targeted training and educational programs including new and more detailed educational clinical vignettes relevant for non-direct access countries are needed to enhance physiotherapists’ diagnostic skills and decision-making processes.

**Supplementary Information:**

The online version contains supplementary material available at 10.1186/s12875-024-02634-8.

## Introduction

Musculoskeletal (MSK) conditions are highly prevalent within the general population [1]. Low back pain (LBP), for instance, is the leading cause of years lived with disability for men in 133 and in 104 countries for women world-wide [2]. The vast majority of MSK conditions are of a benign nature, do not require highly specialized and/or surgical attention, and can be managed in a primary care setting [1, 3, 4].

Moreover, we are living in an ageing society with the proportion of people aged 65 years and older predicted to increase from 34.4 to 59.2% over the next five decades [5]. Advanced age is associated with a higher incidence of musculoskeletal conditions and a higher risk of developing a serious pathology [6]. The change in proportion of older age groups in our societies may lead to an increase in health care costs [5] due to the potential rise in people developing serious musculoskeletal conditions and serious pathologies. There is a clear link between advanced age and serious pathology affecting the MSK system such as osteoporosis [7] and many types of cancers such as prostate, lung, bowel and breast [8, 9]. For example, 80% of all fractures in women over 50 years old are linked to osteoporosis [7], and prostate, lung and breast cancer are one of the main causes of all osseous metastases [10]. An ageing society imposes challenges to healthcare systems to cope with the rise in demand and timely detection of serious pathologies. Primary Care has been advocated as one approach to cope with these challenges, with World Health Organisation [11] endorsing it is a clinically and cost-effective approach to enhance people’s physical and mental wellbeing. Based upon the benefits offered by primary care, in Austria, the number of primary care units is expanding [12]. Their core principle being that patients are managed in a team consisting of general practitioners and other healthcare professionals such as nurses, physiotherapists, speech and language therapists and occupational therapists [12]. Despite the development of primary care units in Austria, all patients still need to have been referred by a medical doctor to see a physiotherapist [13]. Therefore, Austria does not have a direct access system. In addition, training for specialisations, such as ‘specialised musculoskeletal physiotherapist’, are not yet established in Austria.

Early detection of a serious pathology is essential as this significantly improves prognosis and outcome [14]. However, it is also well known that early recognition is challenging [14] as in the prodromal disease stage there are few non-specific signs and symptoms of a serious pathology [15]. It is only as the disease progresses, that these signs and symptoms become more obvious. Physiotherapists usually work over prolonged periods with their patients, and as such, are well placed to monitor and screen for specific signs and symptoms (red flags) which might indicate the presence of a serious pathology [16].

The following approach of ‘keep’; ‘refer’ or ‘keep and refer’, has been proposed to provide a framework to guide clinical reasoning and inform the management of musculoskeletal conditions and serious pathologies. It is a core element in the World Confederation of Physical Therapists’ Guideline for Standards of Physical Therapy Practice [17]. ‘Keep’ is where a patient’s condition is suitable for physiotherapy management. ‘Refer’ is where a patient’s condition is not suitable for physiotherapy management and needs referral to a medical doctor. ‘Keep and refer’ is where the physiotherapist can treat the patient with additional medical evaluation [16, 18–21]. However, international cross-sectional studies using this approach with qualified physiotherapists [18, 21–31], Doctor of Physical Therapy (DPT) students [32], and European final year undergraduate physiotherapy students [20, 33] have raised concerns about physiotherapists’ and physiotherapy students’ ability to identify serious pathology and their clinical reasoning to determine if physiotherapy management is indicated, or not.

It has been reported that physiotherapists were only able to recognise between 40 and 79% of the clinical vignettes pertaining to critical cases, which needed immediate referral to a medical doctor [21, 22, 25, 29, 32, 34].

In cognisance of the changes to Austrian healthcare, which places physiotherapists as a core profession assessing patients in the primary care setting, it is key to explore the ability of registered Austrian physiotherapists to accurately detect serious pathologies and to determine if a patient is suitable for physiotherapy (keep), or rather needs a more comprehensive medical examination (refer).

Our main aim was to assess the ability of registered Austrian physiotherapists to make accurate keep-refer decisions to detect the presence of serious pathology affecting the MSK system based on 12 clinical vignettes [18]. Our secondary aim was to investigate the need for red flags education under registered Austrian physiotherapists by assessing confidence in their keep-refer decisions, the relevance of the existing clinical vignettes, and the interest in continued education regarding red flags by registered Austrian physiotherapists.

## Methods

This was an online cross-sectional survey reported in accordance with the Checklist for Reporting Results of Internet E-Surveys (CHERRIES) [35]. A detailed description of the methods has been reported in W Lackenbauer, S Gasselich, ME Lickel, R Beikircher, C Keip, F Rausch, M Wieser, J Selfe and J Janssen [36].

### Human ethics and consent to participate

The study was formally examined by the Commission for Scientific Integrity and Ethics of the Karl Landsteiner Private University, and it was found that in accordance with the set criteria a further review by the commission was not required (Project nummer:1021/2022, date 20-04-2022). There were no medical ethical concerns about the conduct of the study. All interested physiotherapists needed to provide consent to participate.

### Eligibility criteria

To be eligible, participants needed to be registered as physiotherapists with the Austrian Health Professions Registry and work in a hospital and/or private setting in Austria. Registered Physiotherapists who had not been actively treating patients during the last 12 months were not eligible.

### Recruitment

All physiotherapists who were, at the time of the study, registered with the Austrian Health Professions Registry as a self-employed practitioner and had a publicly available email address were contacted. On the 10th of May 2022, an email with an explanation of the study and a link to take part in the survey was sent. A reminder was sent two weeks after the initial invitation to boost recruitment [37, 38].

To reach registered physiotherapists working in a hospital, rehabilitation setting or other health institution, we sent the multidisciplinary team leaders of 44 health institutions in Austria an email with information about the study and asked to provide information on the number of registered physiotherapists currently working for the institution. For those who agreed to take part in the study, a new email was sent on the 7th of June 2022 containing a link for the survey to be forwarded to the registered physiotherapists. A reminder was sent two weeks after the initial invitation to maximise recruitment.

### Sample size calculation

On the 15th of March 2022, 16,991 physiotherapists were registered on the Austrian Health Professions Registry. Given this population, 376 registered physiotherapists were needed to complete the survey to retain a confidence interval of 95% with a margin of error of 5% [39].

### Survey

We used the survey tool Unipark [40]. The survey took approximately 15–20 min to be completed. The first part consisted of questions on demographics, including; age, sex, time working as a physiotherapist, specialisation, additional ‘red flags’ courses, employment status, and educational level.

Following the questions on demographics, participants answered questions based upon 12 previously tested clinical vignettes [18], translated from English to German. Participants had a maximum of 15 min to complete the 12 vignettes, which is in line with previous studies [20, 22]. The 12 vignettes were taken from CR Budtz, H Rønn-Smidt, JNL Thomsen, RP Hansen and DH Christiansen [18] (with permission) and detailed information about the reasoning for the categorisation plus the specific diagnosis, where applicable, are accessible in the supplementary section in the original publication [18]. Participants could only provide one answer per clinical vignette: keep; keep and refer; or refer. The clinical vignettes were divided into musculoskeletal (*n* = 5), non-critical medical (*n* = 4), and critical medical (*n* = 3) categories following the original classification [18, 20–22, 28, 29, 32]. Musculoskeletal vignettes described conditions, which would be appropriate for the physiotherapist to manage without consulting the patient’s medical doctor. A correct answer for the musculoskeletal vignettes was to treat the patient without the need for medical referral (keep) or to treat the patient with additional medical evaluation (keep and refer). Non-critical medical described conditions, which should not be treated by a physiotherapist alone, therefore a correct answer for these vignettes was to start physiotherapy with additional medical evaluation (keep and refer) or refer the patient without physiotherapy management (refer). Critical medical described conditions, which should not be treated by a physiotherapist. The sole correct answer for these vignettes was the decision to send the patient for medical evaluation without physiotherapy management (refer).

After completing the clinical vignettes, participants were also asked to provide feedback about the need for red flags education. Physiotherapists’ level of confidence in decision making skills and relevance of the vignettes to daily practice were measured with Likert scales (1–4; very confident to not confident, very relevant to not relevant respectively). In addition, participants were asked with yes/no questions if they had received enough education on red flags during their physiotherapy career, and if they were interested in learning more about keep-refer decision making.

### Pilot testing

The first version of the survey was piloted with a panel of five registered Austrian physiotherapists (four with experience in musculoskeletal health and one in internal medicine) and four medical doctors (one with experience in internal medicine, in orthopaedics, one in oncology and one master student of medicine) (Portney and Watkins, 2015). Members of this panel were asked to comment on (i) their general understanding of the questionnaire, (ii) the appropriateness, comprehensibility as well as proper sequencing of individual questions and (iii) if any additional questions were deemed valuable. For instance, one question was added in the survey to inquire if they had been treating patients within the last 12 months. If the physiotherapist answered ‘no’, they were thanked for their interest and then automatically prevented from continuing with the survey. The validity of some of the vignettes in the Austrian health care system was also questioned by one member of the pilot testing panel. We decided to replace the original vignettes by DU Jette, K Ardleigh, K Chandler and L McShea [21] by the more recent vignettes of CR Budtz, H Rønn-Smidt, JNL Thomsen, RP Hansen and DH Christiansen [18].

### Data management and analysis

The survey was online and progression through the 12 vignettes was programmed so that an answer was mandatory before progressing to the next vignette. Data collected in the survey was automatically stored at the QuestBack server park in Bremen, Germany. Only when all 12 clinical vignettes had been completed, the data was included in the analysis. After data collection was completed, raw quantitative data was downloaded to an SPSS file (IBM SPSS Statistics 28.0). All data was stored on a secure OneDrive folder to which only the researchers had access through their password protected laptops. The survey was designed to be anonymous, however there was a small risk that participants could share personal data in the open-ended question in the last section of the survey. If this happened data were anonymised by a member of the research team (SG).

Response rate could only be calculated for the self-employed physiotherapists as only the self-employed physiotherapists were invited individually.

All quantitative data was checked for completeness and outliers. Demographic data was then analysed descriptively using frequency and percentages. In order to calculate the ability of physiotherapists to make accurate keep-refer decisions, the mean percentages of correct keep-refer decisions of each category (MSK, medical non-critical or medical critical) and the percentages of 100% correctly answered categories were calculated [18]. To assess the need for red flags education the data was analysed descriptively using frequency and percentages.

## Results

The survey was distributed to 5164 self-employed registered physiotherapists and 44 multidisciplinary team leaders between May 2022 and June 2022. In total 561 physiotherapists fitted the in- and exclusion criteria and consented for participation in the survey. 479 (85.4%) participants fully completed all the clinical vignettes and were included in the analysis. Of the 82 participants who did not complete the clinical vignettes, most stopped at the introduction of the clinical vignettes (*n* = 13, 15.9%) or at the first clinical vignette (*n* = 14, 17.1%). The response rate from the direct emails to the self-employed physiotherapists was 8.0%. The anonymised results of the questionnaire are available at Github: https://github.com/JcadJanssen/redflagsurvey.

The majority of the participants (62.2%) were self-employed, identified as female (70.6%) and had over 20 years of clinical experience (33.4%). The characteristics of the participants are presented in Table 1.

**Table 1:** Descriptive statistics of the participants

	*N*	%
Number of physiotherapists	479	100
Gender		
Woman	338	70,6
Man	131	27,3
Non-binary	2	0,4
Missing	8	1,7
Age (years)		
20–25	16	3,3
26–30	71	14,8
31–35	73	15,2
36–40	93	19,4
41–50	134	28,0
51–60	80	16,7
> 60	10	2,1
Missing	2	0,4
Clinical experience (years)		
0–5	75	15,6
6–10	94	19,6
11–15	76	15,9
16–20	74	15,4
> 20	160	33,4
Specialty		
Musculoskeletal	355	74,1
Neurology	54	11,3
Geriatrics	28	5,8
Cardiorespiratory	22	4,6
Gynaecology	19	4,0
Missing	1	0,2
Additional qualifications		
Yes	144	30,1
No	315	65,8
Missing	20	4,2
Employment setting		
Self-employed	298	62,2
Employed	62	12,9
Employed and self-employed	118	24,6
Missing	1	0,2
Level of education		
PhD	5	1,0
Master	84	17,5
Bachelor	151	31,5
Diploma	225	47,0
Missing	14	2,9

### Decision making skills

Only 2 (0.4%) participants answered all 12 vignettes correctly (see Table 2). The average percentage of correct answers for the MSK, non-critical medical (NCM) and critical medical (CM) categories were 70.5, 79.4 and 53.5 respectively. Vignette 10 in the MSK category and 9 in the CM category had the lowest accuracy rates.

**Table 2:** Overview of the average mean scores and 100% correct cases of the three categories

Vignette cases*	Keep *n* (%)	Keep & refer *n* (%)	Refer *n* (%)	Correct answers *n* (%)	100% correct cases *n* (%)
**Musculoskeletal**				**70.5 (mean)**	**54 (11**,**3)**
Case 3: Man with leg pain	158 (33.0)	251 (52.4)	70 (14.6)	409 (85.4)	
Case 4: Woman with neck pain	324 (67.6)	136 (28.4)	19 (4.0)	460 (96.0)	
Case10: Woman with pain around sternum	17 (3.5)	74 (15.4)	388 (81.0)	91 (19.0)	
Case 8: Girl with knee pain	60 (12.5)	210 (43.8)	209 (43.6)	270 (56.4)	
Case 6: Man with knee pain	227 (47.4)	232 (48.4)	(20) 4.2	459 (95.8)	
**Non-critical medical**				**79.5 (mean)**	**177 (37.0)**
Case 1: Man with bilateral leg cramps	46 (9.6)	292 (61.0)	141 (29.4)	433 (90.4)	
Case 2: Woman with foot pain	218 (45.5)	177 (37.0)	84 (17.5)	261 (54.5)	
Case 7: Woman with bilateral shoulder pain	118 (24.6)	219 (45.7)	142 (29.6)	361 (75.4)	
Case 11: Woman with intense subcostal pain	12 (2.5)	82 (17.1)	385 (80.4)	467 (97.5)	
**Critical Medical**				**53.5 (mean)**	**68 (14.2)**
Case 5: Man with swollen and red knee	25 (5.2)	180 (37.6)	274 (57.2)	274 (57.2)	
Case 9: Woman with intense low back pain	162 (33.8)	186 (38.8)	131 (27.3)	131 (27.3)	
Case 12: Man with thoracic back pain	15 (3.1)	100 (20.9)	364 (76.0)	364 (76.0)	
**Total**				**69.2 (mean)**	**2 (0.4)**

*Vignettes cases available at Budtz et al. (2021)

### Need for red flags education

Two in three (67%) participants felt confident/very confident screening for red flags (Table 3). 344 (71.8%) of the participants reported learning through clinical vignettes as relevant/very relevant (Table 4).

**Table 3:** Confidence of registered Austrian physiotherapists when answering the clinical vignettes

Level of confidence	*n* (%)
Very confident	23 (4.8)
Confident	298 (62.2)
Limited confidence	120 (25.1)
Not confident	18 (3.8)
Missing	20 (4.2)

**Table 4:** Clinical relevance of the clinical vignettes for registered Austrian physiotherapists

Clinical relevance	*n* (%)
Very relevant	93 (19.4)
Relevant	251 (52.4)
Limited relevance	108 (22.5)
Not relevant	20 (4.2)
Missing	7 (1.4)

279 (58.2%) of the participants stated that they had not received enough information regarding red flag screening during their bachelor’s degree. In comparison, 137 (28.6%) thought red flags screening was sufficiently covered. 378 (78.9%) of the participants expressed interested in continuing their training in red flags.

## Discussion

This was the first study to examine the keep/refer decision making abilities of registered Austrian physiotherapists based on concise clinical vignettes. A total of 479 registered Austrian physiotherapists completed the survey. A correct decision for 100% of the vignettes for the MSK, the medical non-critical and medical critical category was made by 11.3%, 37.0% and 14.2% of the participants, respectively. On average, participants answered 70.5% of the MSK vignettes correctly, while for the medical non-critical and medical critical vignettes, a correct decision was made in 79.4% and 53.5%, respectively. Compared to previous studies using similar [20–22, 25, 29, 32, 33] or identical vignettes [18], registered Austrian physiotherapists scored below the average in each of the three categories. The results for the medical critical category were not entirely surprising as 59% of responding physiotherapists felt that screening for serious pathology (Red Flag Screening) was not sufficiently covered in their education. A lack of formal training, a lack of knowledge about individual differential diagnoses and specific guidance on how to recognize the presence of a serious pathology has already been voiced by qualified physiotherapists in Denmark [41]. The results of the current study also highlight the need for additional, specialized training on how to make keep/refer decisions and in recognizing the presence of serious pathology for registered Austrian physiotherapists. Previous studies have already demonstrated that supplementary in-depth red flag training is effective in improving the ability of physiotherapists to make accurate keep/refer decisions and recognise the presence of serious pathology [42–44]. In addition, study participants from CR Budtz, H Rønn-Smidt, JNL Thomsen, RP Hansen and DH Christiansen [41] indicated that feedback from doctors, to whom they had referred patients back for further evaluation, could help them to further develop their differential diagnostic skills. One of the aims of the expansion of primary care centres in Austria is to strengthen interprofessional cooperation and communication [12]. In the future, this planned close exchange could help Austrian physiotherapists to benefit from the differential diagnostic skills of MDs and thus improve their own skills. Even though the clinical vignettes used in the current study had been by expert physiotherapists and medical doctors [18, 21], controversy remains about the most appropriate decision in some of the vignettes. The results of this study show that in some clinical vignettes (MSK vignette 10, CM vignette 9) the majority of participants (81% and 72.7% respectively) did not answer the vignettes correctly.

In MSK vignette number 10, which describes a clinical picture of costochondritis [18, 21], the correct answers to a MSK vignette are either “keep” or “keep and refer”. However, 81% of the current study sample chose to refer this patient to a medical doctor without any physiotherapy involvement. This decision is in line with that of the emergency doctor who took part in the validation process of the clinical vignettes from a medical perspective for the study of DW Vaughn, MJ Shoemaker, D Da Prato, KS Murray and J Van Huisen [32]. They argued that a clinical situation as described in this vignette could also very likely indicate a cardiac pathology and therefore must be regarded as a medical emergency. Interestingly this vignette also consistently recorded the lowest percentages of correct answers in the MSK category in previous studies [18, 21, 22, 29, 32].

In this context, the results of vignette number 9 also need to be discussed. This vignette describes a clinical picture with several risk factors, signs and symptoms indicative of a vertebral fracture [45, 46]. Only 15% of the study participants in CR Budtz, H Rønn-Smidt, JNL Thomsen, RP Hansen and DH Christiansen [18] and 27% of participants in our study made the correct decision (refer without physiotherapy). Alarmingly, more than half of Danish physiotherapists [18] and 33.8% in our study chose to start physiotherapy without considering at least additional medical referral.

### Limitations

In the preparation of the study the aim was to collect the email addresses of all registered physiotherapists currently registered to work in Austria. Unfortunately, from the self-employed registered Austrian physiotherapists not all email addresses were retrievable online and 50% of the population could not be contacted.

The limitations of clinical vignettes as a sole instrument for examining clinical decision-making strategies of health care professionals have previously been highlighted [47–49]. In addition, other authors [18, 22] have already highlighted that physiotherapists usually collect more detailed patient data, background information and findings from the physical examination to make clinical decisions is included in these short vignettes. However, vignettes have the advantage that they can be distributed to a large pool of potential participants relatively quickly and at low cost.

Another limitation pertains to the validity of the vignettes used in this study. Whilst the clinical vignettes of DU Jette, K Ardleigh, K Chandler and L McShea [21] went through a content validation, the clinical vignettes by CR Budtz, H Rønn-Smidt, JNL Thomsen, RP Hansen and DH Christiansen [18] were passed through a consensus method. This might have influenced the validity of the used vignettes. However, the research team chose the use the clinical vignettes by CR Budtz, H Rønn-Smidt, JNL Thomsen, RP Hansen and DH Christiansen [18] as it was felt they reflected the clinical practice of physiotherapy in Austria better than the clinical vignettes by DU Jette, K Ardleigh, K Chandler and L McShea [21].

Non-response bias could have played a factor in this study. Similar studies [21, 32] concluded that possibly only clinicians who felt competent enough to make an accurate keep/refer decision completed their surveys.

C Beyerlein [22] has already highlighted a potential problem with the answer option ‘keep and refer’ in certain clinical scenarios. His critique is understandable, as the question arises as to what additional role physiotherapy plays in a suspected stress fracture in the metatarsal region [18] apart from sending the patient to a MD for further evaluation. However, to enable comparison with previous similar studies, the three different answer option have been retained.

Finally, the generalisability of our findings should be treated with caution due to the small number of Austrian physiotherapists surveyed.

## Conclusion

This was the first study to examine the keep/refer decision making abilities of Austrian qualified physiotherapists based on internationally clinical vignettes. Based upon the overall low accuracy rates of the correct decision for the vignettes and the indication from participants that further training is needed, our findings suggest that there is scope for additional education content to be created for qualified Austrian physiotherapists. The findings of this study are also a precursor to further research focusing on the education of Austrian physiotherapists in relation to screening for serious pathology, including inter-professional communication and collaboration, the development and implementation of specific guidelines and referral pathways for all health professionals (including physiotherapists) and the potential impact of specialisation, for example musculoskeletal, in physiotherapy.

## Electronic supplementary material

Below is the link to the electronic supplementary material.

Supplementary Material 1

## Data Availability

The anonymised data of the questionnaire are available at Github: https://github.com/JcadJanssen/redflagsurvey.

## Abbreviations

MSK: Musculoskeletal
LBP: Low back pain
DPT: Doctor of Physical Therapy
CHERRIES: Checklist for Reporting Results of Internet E-Surveys
NCM: Non-critical medical
CM: critical medical
