# Supplementary material for: The ability of Austrian registered physiotherapists to recognize serious pathology
Source: BMC Prim Care. 2024 Oct 30;25:387. doi: 10.1186/s12875-024-02634-8 (PMC11523903; doi:10.1186/s12875-024-02634-8)
Supplement: Supplementary file 1 — Supplementary Material 1 [file 12875_2024_2634_MOESM1_ESM.pdf]

Red flags questionnaire

1. **Demographic Data**

How old are you?			
20-25	26-30	31-35	36-40
41-50	51-60	>60	No answer

Gender			
Woman	Man	Non-binary	No answer

How many years have you been working as a physiotherapist?			
1 year	2-3 year	4-5 year	6-10 year
11-15 year	16-20 year	>20 year	No answer

In which field do you primarily work?		
Musculoskeletal (orthopaedics, traumatology, sports, osteopathy)	Neurology, paediatrics	Geriatrics, psychiatry
Cardiorespiratory (cardiology, pulmonology, internal medicine [metabolism])	Gynaecology, oncology, occupational medicine/health promotion	

Have you taken part in at least one course focussing on ‘Red Flags’?		
Yes	No	No answer

Are you working as a private practice physiotherapist (self-employed) or are you employed?			
Self-employed	Employed	Self-employed and employed	No answer

What is your highest level of education in the field of physiotherapy?		
PhD	Master	Bachelor
Diploma	No answer	

2. Introduction Clinical vignettes (CV)

12 clinical vignettes (CVs) are presented below. Due to time constraints, the following CVs contain the most important patient information needed to make a decision. You have a time limit of 15 minutes to answer all the CVs.

There are 3 possible answers available:

- Physiotherapy treatment
- Physiotherapy treatment and additional GP referral
- Direct GP referral and no physiotherapy treatment

Only 1 answer can be chosen per CV.

By clicking on 'Next', the 15-minute timer starts and the CVs can be worked through one after the other. The questions must be completed in the correct order; no question can be repeated.

3. Clinical vignettes from Budtz et al. 2021

**MUSCULOSKELETAL CONDITIONS**		
**Nr**	**Case**	**Clinical vignette**
**3**	Man with leg pain	A 45-year-old man complains of consistent pain in his right leg, especially around the thigh. He is a builder and have had heavy and physically demanding work for the past 25 years. In the past couple of years, he has had episodes of low back pain. The pain had a sudden onset, but he does not recall a precipitating incident or injury. ROM is decreased in the lower back, especially in flexion to the right side which also aggravates the pain.
**4**	Woman with neck pain	A 39-year-old woman complains of 6-9 months of intermittent but increasing pain and tenderness around the neck area. There is local muscular tenderness and pain with movement. ROM of the neck is slightly decreased. There is no dizziness or radiation of pain.
**6**	Man with knee pain	A 65-year-old man, former football player and current tennis player, complains of pain in his left knee, worsening over the past 6 months. The pain is affecting his leisure time activities as it increases with activity and movement. He reports a feeling of grinding in his knee. No swelling is noted, and the ROM is normal.
**8**	Girl with knee pain	A 17-year-old girl complains of knee pain following a football game where she stepped into a hole. She was unable to complete the remainder of the game. The medial aspect of the knee is tender to palpation and slightly swollen. The pain increases at the ends of ROM and with valgus stress. No complaints of knee locking.
**10**	Woman with pain around sternum	A 53-year-old woman with a fairly sedentary lifestyle complains of a sudden onset of deep and intense pain around the sternum. The pain increases with movement of the left arm. She cannot identify a precipitating incident or injury. The pain is not radiating, and there is extreme tenderness to palpation lateral to the sternum. Coughing and sneezing increase the pain.

NON-CRITICAL MEDICAL CONDITIONS		
**1**	Man with bilateral leg cramps	A 65-year-old man with a history of COPD and significant cigarette smoking complains of bilateral leg cramping associated with stair climbing beginning about 6 months ago. Over the past 2 months, the cramping has become associated with walking as well. When he sops walking and stands still, the cramping decreases and then disappears. No reflex or sensory changes are detected, and the pain is not affected by trunk flexion er extension.
**2**	Woman with foot pain	A 35-year-old woman who is healthy and an occasional jogger complains of pain in the anterolateral aspect of the forefoot starting about a week ago. The pain started when she was running and is exacerbated by any weight bearing. There is moderate tenderness to palpation; no swelling or redness is noted. Foot and ankle ROM are normal.
**7**	Woman with bilateral shoulder pain	A 63-year-old woman complains of bilateral shoulder pain and stiffness. The pain has been increasing over the past months, and she feels it is worsening. She cannot identify a precipitating incident or injury. The pain typically decreases during the day. There is bilateral muscle weakness and limited ROM.
**11**	Woman with intense subcostal pain	A 55-year-old woman complains of constant, intense aching back pain subcostally on the right side over the past 2-3 days. The pain radiates along the iliac crest on the right side. She cannot identify a precipitating incident or injury. The pain is not affected by positional changes or the use of a heating pad.
**CRITICAL MEDICAL CONDITIONS**		
**5**	Man with swollen and red knee	A 60-year-old woman who apparently is healthy, but inactive, complains of sudden onset of pain in the right knee with no known precipitating incident. The joint is very tender, warm, and red. ROM is painful and decreased. No other previous or current joint complaints are reported.
**9**	Woman with intense low back pain	A 75-year-old woman complains of intense low back pain. The pain started while she was moving heavy boxes around. However, she cannot identify a precipitating incident or injury. The pain decreases with rest and is not radiating. There is extreme localized tenderness to palpation.
**12**	Man with thoracic back pain	A 45-year-old man complains of mild-to-moderate thoracic back pain that is preventing his sleeping at night. The pain is intermittent but has increased over the past 2 weeks and is not relieved by positional changes. It seems to be worse at night than during the day. The patients complain of fatigue that he attributes to not sleeping well.
Abbreviations: COPD = Chronic Obstructive Pulmonary Disease, ROM = Range of Motion, Budtz et al 2021		

4. **Questions to the CVs**

Thank you for answering the CVs!

How confident did you feel in answering them?		
Very confident	Confident	Little confidence
Not confident	No answer	

How relevant are these CVs to your daily work?		
Very relevant	Relevant	Limited relevance
Not relevant	No answer	

What relevant information was missing in the CVs to make a clinical decision? (open ended question)

5. **Educational tool**

Do you have the impression the subject 'Red Flag Screening' has been sufficiently covered during your education or as part of further specialised training?		
Yes	No	No answer

Are you interested in further training on 'Red Flag Screening'?		
Yes	No	No answer

In which setting or with which medium could you imagine further training in this area? (multiple answers possible)			
One-day workshop in a face-to-face setting	Multi-day workshop in a face-to-face setting	Online seminars	Hybrid Workshop (online and face-to-face)
App	Literature (books + articles)	Website	Other

How should further training be specifically organised? (multiple answers possible)		
Clinical Vignettes in paper form	Clinical Vignettes in videos	Literature on current research
Discussion/exchange	Practical assessment sessions	Other

Feel free to leave further comments in the text field below (open ended question):

6. End

Thank you for participating!

If you are interested in the results of the clinical vignettes, please contact us by e-mail: redflags@…..

We will be happy to send you the results after the end of the survey.
